# Biosensors toward behavior detection in diagnosis of alzheimer’s disease

**DOI:** 10.3389/fbioe.2022.1031833

**Published:** 2022-10-19

**Authors:** Xiaotong Sun, Xu Sun, Qingfeng Wang, Xiang Wang, Luying Feng, Yifan Yang, Ying Jing, Canjun Yang, Sheng Zhang

**Affiliations:** ^1^ Ningbo Innovation Center, School of Mechanical Engineering, Zhejiang University, Ningbo, China; ^2^ Faculty of Science and Engineering, University of Nottingham Ningbo, Ningbo, China; ^3^ Nottingham Ningbo China Beacons of Excellence Research and Innovation Institute, University of Nottingham Ningbo, Ningbo, China; ^4^ Nottingham University Business School China, University of Nottingham Ningbo China, Ningbo, Zhejiang, China; ^5^ Business School, NingboTech University, Ningbo, China

**Keywords:** biosensors, alzheimer’s disease, diagnosis, behavior detection, elderly

## Abstract

In recent years, a huge number of individuals all over the world, elderly people, in particular, have been suffering from Alzheimer’s disease (AD), which has had a significant negative impact on their quality of life. To intervene early in the progression of the disease, accurate, convenient, and low-cost detection technologies are gaining increased attention. As a result of their multiple merits in the detection and assessment of AD, biosensors are being frequently utilized in this field. Behavioral detection is a prospective way to diagnose AD at an early stage, which is a more objective and quantitative approach than conventional neuropsychological scales. Furthermore, it provides a safer and more comfortable environment than those invasive methods (such as blood and cerebrospinal fluid tests) and is more economical than neuroimaging tests. Behavior detection is gaining increasing attention in AD diagnosis. In this review, cutting-edge biosensor-based devices for AD diagnosis together with their measurement parameters and diagnostic effectiveness have been discussed in four application subtopics: body movement behavior detection, eye movement behavior detection, speech behavior detection, and multi-behavior detection. Finally, the characteristics of behavior detection sensors in various application scenarios are summarized and the prospects of their application in AD diagnostics are presented as well.

## 1 Introduction

By 2020, more than 50 million people suffer from dementia globally, and this number will keep increasing at a high rate, rising to 82 million in 2030 and 152 million in 2050. Among them, developing countries will account for the majority of the growth. By 2050, 71% of dementia patients would be residents of low- and middle-income nations, up from the current 60% (Prince et al., 2015). Alzheimer’s disease, the most prevalent cause and well-known type of dementia, accounts for 60–80% of incidences (Gauthier et al., 2021). Currently, there is no effective pharmacological medication that can halt the progression of the pathology, making early diagnosis and intervention extremely crucial (Mancioppi et al., 2019). Therefore, the early diagnosis of AD is becoming an important issue and is attracting increasing attention worldwide. However, the existing main techniques used to accurately diagnose AD include Cerebrospinal fluid (CSF) testing and neuroimaging methods, which are costly, time-consuming, and out of reach for the majority of people. In this context, biosensors are considered as potential substitutes for low-cost, quick, comfortable, and straightforward method for AD diagnosis (Brazaca et al., 2019).

Biosensors are analysis tools that transform biological responses into quantifiable signals (Amine et al., 2006; Brazaca et al., 2019), they have significant potential to revolutionize a wide range of industries, especially are extensively utilized in the field of medical testing, such as health monitoring, motion detection, activity identification, *etc.* (Liu et al., 2016; Sprint et al., 2016; Petra et al., 2018). Among them, the physiological biosensor is one of the most common categories that are utilized to detect biochemical indicators, e.g., pH, glucose, ions, sweat, skin interstitial fluid in the human biofluids, and physical indicators, such as pulse, heart rate, blood pressure, temperature, electrocardiogram (ECG), *etc.* (Brazaca et al., 2019; Guo et al., 2020; Kodintsev et al., 2020; Zhang et al., 2021a; Zhang et al., 2021b; Huang et al., 2021; Sheng et al., 2022). For instance, the graphene FET sensors has been developed by Bungon et al. to detect protein biomarker clusterin of Alzheimer’s disease (Bungon et al., 2020). Besides, due to the development of health monitoring technology, behavior detection biosensors, such as motion sensors and sound sensors are being widely used in this field to measure various behavioral biomarkers of humans (Bouchard et al., 2014; Chaccour et al., 2016; Vippalapalli and Ananthula, 2016; Stone and Skubic, 2017; Wang et al., 2017; Nweke et al., 2018; Wang et al., 2019 Lima et al., 2019; Fan et al., 2020; Yu and Wang, 2020), e.g., person’s movement and speech behavior, *etc.* (Yu and Wang, 2020). Moreover, ambient biosensors play an essential role in human activity detection and risk assessment by monitoring environmental conditions, including humidity, radiation, gas, pressure, temperature, *etc.* (Sunny et al., 2020; Karthikeyan et al., 2021).

Among these three basic categories of biosensors, behavioral detection sensors are gaining increasing attention because of numerous demands in society, such as safety, comfort, natural interaction, entertainment, assisted living, *etc.* (Yu and Wang, 2020). For instance, the aging problem urges the demand for home-based health care for the elderly (Merilahti et al., 2015). In order to promote the integration of smart homes with elderly care, Donghwa Shon et al. investigated the possibility of embedding healthcare services into smart homes in a non-invasive manner, and propose medical scenarios that can be applied to each smart home room (Choi et al., 2019). The monitoring of activities of daily living (ADL) using the household sensors and networks have been investigated (Nathan et al., 2018). Fleury et al. propose a support vector machine (SVM)-based ADL recognition mechanism. Lots of sensors such as microphones, contact sensors, infrared (IR) sensors, accelerometer and magnetometer are used to monitor the activities of older persons (Fleury et al., 2010). There are usually more than one member in a family, to identify each of the dwellers, Wang et al. presented a multiuser activity recognition system using wearable audio sensors, altimetry sensors and RFID tags, and high accuracy was obtained (Wang et al., 2011). To ensure they live in a safer environment, behavior monitoring, such as anomalous behavior detection, is urgently needed for elderly people, especially for those living alone (Alemdar and Ersoy, 2010; Rashidi and Mihailidis, 2013; Hoque et al., 2015; Chaccour et al., 2016; Stone and Skubic, 2017; Deep et al., 2020). In addition, biosensor-based behavior detection approaches are also commonly applied to identify behavioral disorders in certain types of diseases, such as Parkinson’s disease (PD), Stroke, Paralysis, *etc.* (Kang et al., 2010; Leclair-Visonneau et al., 2017; Alvarez et al., 2018; Loopez-Larraz et al., 2018; Migliaccio et al., 2020; Borzì et al., 2021; Diaconu et al., 2021). Notably, many recent studies have revealed that behavioral biomarkers might be vital indicators of Alzheimer’s disease (AD) in its initial phases (de et al., 2014; Nardone et al., 2022; Staal et al., 2021), symptoms like motor behavior alteration occur before profound memory deficits (Tippett and Sergio, 2006; Casper et al., 2015). Thus, given the fact that motor behavior deficits are generally regarded as a consequence of aging (Tippett and Sergio, 2006), biosensor-based behavior detection is vital for both the early diagnosis of AD, and for assisting to explore the underlying causes of the daily activity dysfunction of the elderly in a quantitative way. Therefore, this objective and effective approach to detecting the behavior biomarkers of potential AD patients by using biosensors is becoming a promising diagnostic method for AD (Verheij et al., 2012; Abe et al., 2013; Alam et al., 2017). As a result, a variety of wearable and ambient non-invasive biosensors for AD diagnosis are emerging (Wams et al., 2017; Mancioppi et al., 2019). Especially, biosensors oriented to motion behavior monitoring, physiological signal capturing, and environment information detecting have drawn particular attention among AD diagnosis and other behavior detection applications (Lara and Labrador, 2013; Bulling et al., 2014; Liu et al., 2021).

In this review, the most recent research into biosensors for behavior detection in the diagnosis of Alzheimer’s disease is discussed. An electronic database search was performed using the Web of Science, PubMed, Elsevier Science Direct, Scopus R, and Biomed Central databases to identify and select articles concerning the early diagnosis of Alzheimer’s disease using biosensors. The following key words are used: Alzheimer’s disease, diagnosis, biosensor, Behavior. Repetitive articles are eliminated, articles published by the same author are compared, and the articles most relevant to the theme of this article are selected. The applications of behavior detection biosensors are categorized into four groups: body motion behavior detection of Alzheimer’s disease, eye movement behavior detection of Alzheimer’s disease, speech and language behavior detection of Alzheimer’s disease, and multimodal behavior detection of Alzheimer’s disease. Body motion behavior sensors can detect body motions, for example, gait, walking speed, walking stride, balance, foot kicking, upper limb motion, and other activities of daily living. Eye movement behavior sensors are employed to measure gaze, fixation, saccade, eye blinks, and pupil response metrics, *etc.* Speech and language behavior sensors are mainly used to record and identify acoustic features. While multimodal behavior sensors can sense multiple combined behavior parameters, e.g., physiological indicators, eye movement, sound, body motions, *etc.* Together with various environmental information. Moreover, this review provides an outlook on future research directions of biosensor-based behavior detection technology and methodology for the diagnosis of Alzheimer’s disease. Notably, the challenges of data security, quality and privacy, and the regulatory requirements are still exist. Therefore, ethical review must be done prior to testing on patients, patient consent must be sought before data collection can take place, and data involving privacy cannot be published without patient consent.

## 2 Applications of biosensors toward behavior detection of alzheimer’s disease

### 2.1 Body motion behavior detection of alzheimer’s disease

Body motion behavior has been widely studied in detecting AD based on a range of sensors (Robben et al., 2016). Those utilized biosensors are mainly wearable sensors, including feet and waist-mounted inertial sensors, ankle-mounted accelerometric sensors, wrist-mounted accelerometers, leg-mounted force sensors, *etc.* In addition, to realize real-time monitoring and data analysis (Catarinucci et al., 2015; Jain et al., 2021), these biosensor devices tend to be integrated into the internet of things (IoT) devices (Helen et al., 2018; Yamini, 2020; Machado et al., 2021). Besides, with the increasingly improved communication technology, the use of a biosensor to detect body motion behavior in a VR environment offers a more promising way to conduct relevant experiments, which enables subjects to perform the tasks more interactively and safely in a relatively ideal immersive experimental environment (García-Betances et al., 2015; Montenegro and Argyriou, 2017; Mohammadi et al., 2018; Montenegro et al., 2020).

Wearable biosensors are widely employed in body motion detection of AD patients, and most of them are used to detect lower limbs’ motion parameters. Scholars collected body movement data from normal and Alzheimer’s patients, extracted the characteristics of the patient’s movement data and the characteristics of the normal person’s body movement data set to distinguish them with machine learning, and grouped them to determine where the test target belonged. In particular, gait analysis has received great attention in the study of cognitive impairment (Laske et al., 2015). For instance, Hsu et al. suggested a method to conduct objective quantitative measurement of foot movement in AD. In their study, wearable inertial sensor-based devices put on the feet and waist are employed to process gait and balance metrics with the aid of gait and balance analyzing algorithm. The outcomes of the trial demonstrate the effectiveness of this wearable sensor system for early-stage diagnosis of Alzheimer’s disease. It indicates that AD patients present greater variability in gait parameters and worse balance ability than healthy controls (Hsu et al., 2014). In addition, Kirste et al. proposed ankle-mounted three-axes accelerometric sensors to study motion behavior in AD patients’ daily life. All participants are required to conduct a 50 continuous hours’ daily activity recording within 3 days wearing the accelerometric sensor on their ankles. By analyzing the spatial trajectories, subjects’ activity level has been detected. The findings show that this method can discriminate between AD and HC (healthy control) with an accuracy of 91%, and motion behaviors data have a significant correlation with MMSE and CMAI (Cohen-Mansfield Agitation Inventory) scores (Kirste et al., 2014).

**FIGURE 1:**
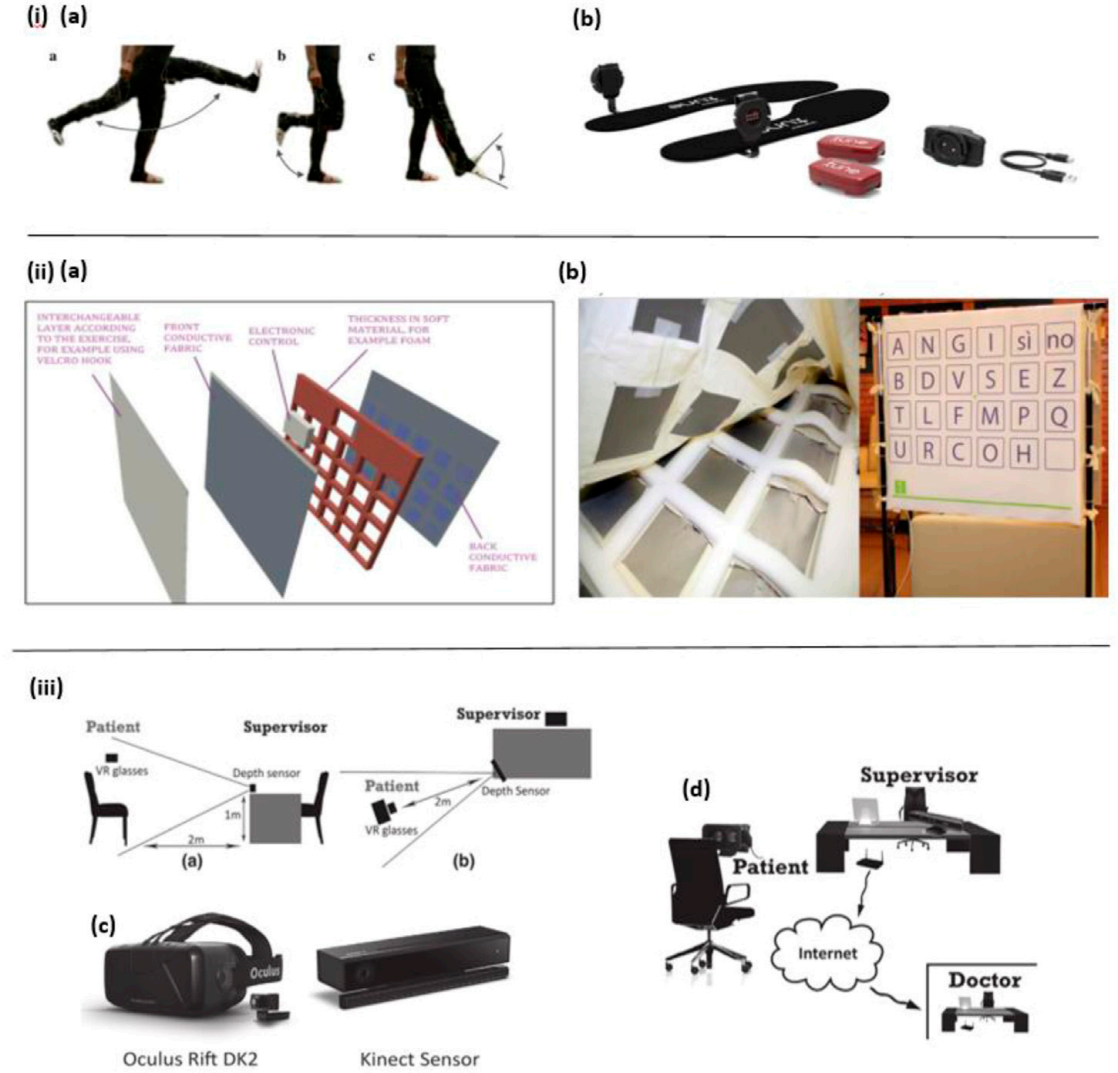
i) **(A)** Leg movement. **(B)** Motion detection device (Varatharajan et al., 2017). ii) **(A)** The SmartTapestry hardware. **(B)** Sensing components in the soft foundation layer (Left) and the SmartTapestry test set-up (Right). (Maselli et al., 2017). iii) **(A)** Side perspective displaying the layout of the experimental set-up. **(B)** Perspective from above displaying the layout of the experimental set-up. **(C)** Virtual reality glasses (Oculus Rift DK2) and depth sensor (Microsoft Kinect 2). **(D)** Early detection setup (Montenegro and Argyriou, 2017).

Varatharajan et al. presented a wearable internet of things (IoT) device to detect early Alzheimer’s disease. As shown in Figure 1I, the force sensor-based leg movement monitoring system is used to collect AD patients’ walking patterns in real-time, and with the help of the dynamic time warping (DTW) algorithm the participants’ various foot movement data, such as walking speed and gait are processed. Moreover, the middle-level cross identification (MidCross) function is applied to classify cognitively normal participants and AD patients by comparing their gait signals. In this study only foot movement is measured, it still proved the effectiveness of this method to diagnose Alzheimer’s disease (Varatharajan et al., 2017).

Moreover, in the work of Fiorini et al. an innovative method of walking behavior detection to diagnose MCI is presented. In this study, aerobic activities are combined with traditional cognitive tools: the TAP test (Test for Attentional Performances), to investigate the feasibility of classifying between MCI and healthy controls. For this reason, the SmartWalk test is adopted to integrate walking behavior and auditory sustained attention by using a wearable 9-axis inertial sensor (a 3-axis accelerometer, a 3-axis gyroscope, and a 3-axis magnetometer), which is fixed on the dominant foot of subjects. As a result, the SmartWalk test shows a positive correlation with the TAP test in cognitive assessment (Fiorini et al., 2017). In another work by Fiorini et al., a similar combined cognitive-physical SmartWalk tool (Sensor foot) is developed, which adds aerobic activity to the traditional cognitive protocols, to explore its correlation with a traditional test in measurement and stimulate cognitive function. In this study, a wider range of subjects has been recruited to participate in the test. Similarly, a wearable 9-axis inertial sensor (a 3-axis accelerometer, a 3-axis gyroscope, and a 3-axis magnetometer) is taped to subjects’ dominant foot to collect walking data. Consequently, the findings suggest that the SmartWalk test is positively associated with the traditional one, and could be useful in cognitive decline intervention (Fiorini et al., 2019).

Besides, daily physical activity behavior has been investigated in some studies. For instance, a wrist-mounted accelerometer is developed in the study of Fleming et al. to record habitual physical activity behavior in non-demented participants with down syndrome (DS). Based on the measured data, the correlation study between participants’ physical activity behavior, cognitive functioning, and imaging biomarkers of Alzheimer’s disease have been conducted. The experimental results revealed that time spent in sedentary behavior is negatively correlated with cognitive functioning, while time spent in moderate-to-vigorous activity behavior correlates with cognitive functioning positively (Fleming et al., 2021). In a similar study by Lu et al., patterns of physical activity and sedentary behavior are compared among participants with AD, MCI, and normal control group in Hong Kong. To obtain these biomarkers a wrist-worn accelerometer has been mounted on every subject’s wrist for 7 days. The results show that AD subjects have longer time sedentary behavior and more sedentary bout than other groups during the day (Lu et al., 2018).

The wearable biosensor offers many advantages, such as portability, comfort, convenience, and allowing for continuous point-of-care testing (Zhang et al., 2021a). At the same time, it has some drawbacks, for instance, some of them are bulky to wear, distracting, and cause tension (Jonell et al., 2021). As a result, in certain cases, some non-wearable biosensors are introduced to create an experimental environment with minimal interference to subjects and maintain the ecological validity of the recorded data, such as ambient-based in-home wireless sensors and Kinect (depth) sensors, *etc.* (Jonell et al., 2021). For example, Maselli et al. designed a sensor-based tapestry to assess and train cognitive functions by combining the measurement of episodic memory and motor (upper limb articulation movement) in the tasks. The SmartTapestry consists of a sensitive base, interchangeable layers, a laptop and a mobile support structure for the tapestry. Based on this device, subjects’ cognitive and motor functions are assessed and trained simultaneously (Figure 1II ). Compared with the traditional approach, this novel tool suggests that the SmartTapestry plays an equivalent role in the assessment and rehabilitation of physical and cognitive function (Maselli et al., 2017).

Furthermore, Urwyler et al. constructed an in-home wireless sensor system, comprising ten unobtrusive senor boxes inside the apartment to detect subjects’ activities of daily living (ADL). These ten sensor boxes were installed in ten different places of the apartment and used to capture light, temperature, humidity, movement and acceleration values with his five ambient sensors. The results show that the recognized ADL data is useful to discriminate between dementia patients and healthy participants with an accuracy of 95% (Urwyler et al., 2017).

Besides, in the study of Fernandez Montenegro et al. a depth sensor is used to track the subjects’ body movements and animate them to improve subjects’ interactivity in the VR environment (Figure 1III). In this way, the subjects wearing the VR glasses can perform the required cognitive tasks in the full-immersive VR environment. In this work, although body behaviors were only recorded and animated, not analyzed and used for diagnosis of AD, it still presents a promising solution for behavior detection in AD diagnosis (Montenegro and Argyriou, 2017).

### 2.2 Eye movement behavior detection of Alzheimer’s disease

Eye movement parameters play a significant role in cognitive function assessment, they can reflect human cognitive and mental state more easily compared to other bio-signals (Zhang et al., 2016), and abnormal viewing behavior has been found among subjects with neurodegenerative conditions in eye movement studies (Anderson and Macaskill, 2013). With the advances in eye-tracking technologies, eye movement parameters, such as gaze, saccade, blink *etc.* Have gained growing interest in various medical fields, including Alzheimer’s disease diagnosis (Crawford et al., 2013; Imaoka et al., 2021). Studies have shown that the brain aggregates information about the position of the eyes and hands in the posterior parietal cortex (PPC), and that anatomical changes in the brain regions of AD patients can lead to damage in the PPC region, which can affect hand-eye coordination tasks. In visuomotor tasks, detecting whether a target’s performance has deficits that are not present relative to age-matched controls provides a preliminary diagnosis of whether the target is ill. On the other hand, specific indicators of the eye and pupil can reflect to some extent the level of cognition, and the analysis of indicators such as the number of gaze durations during visual gaze can also be used to assist in the detection of disease (Skaramagkas et al., 2021).

In many related studies, biosensor-based devices have been constructed in an experimental setup to record eye movement data of AD patients. These biosensors are mainly infrared sensing devices, including wearable devices, e.g., eye-tracking glasses, head-mounted VR eye trackers *etc.*, and non-wearable devices, e.g., desk-mounted eye trackers, handheld pupillometer, infrared eye-tracking camera *etc.* For example, in the work of Fraser et al. a desk-mounted eye tracker is constructed for the measurement of eye-movement features in reading tasks. By analyzing the recorded data, 13 gazes, saccade, and fixation-related features are considered. Ultimately, with the help of machine learning analysis, the healthy control and AD subjects can be distinguished at an accuracy rate of 86% (Fraser et al., 2017).

**FIGURE 2:**
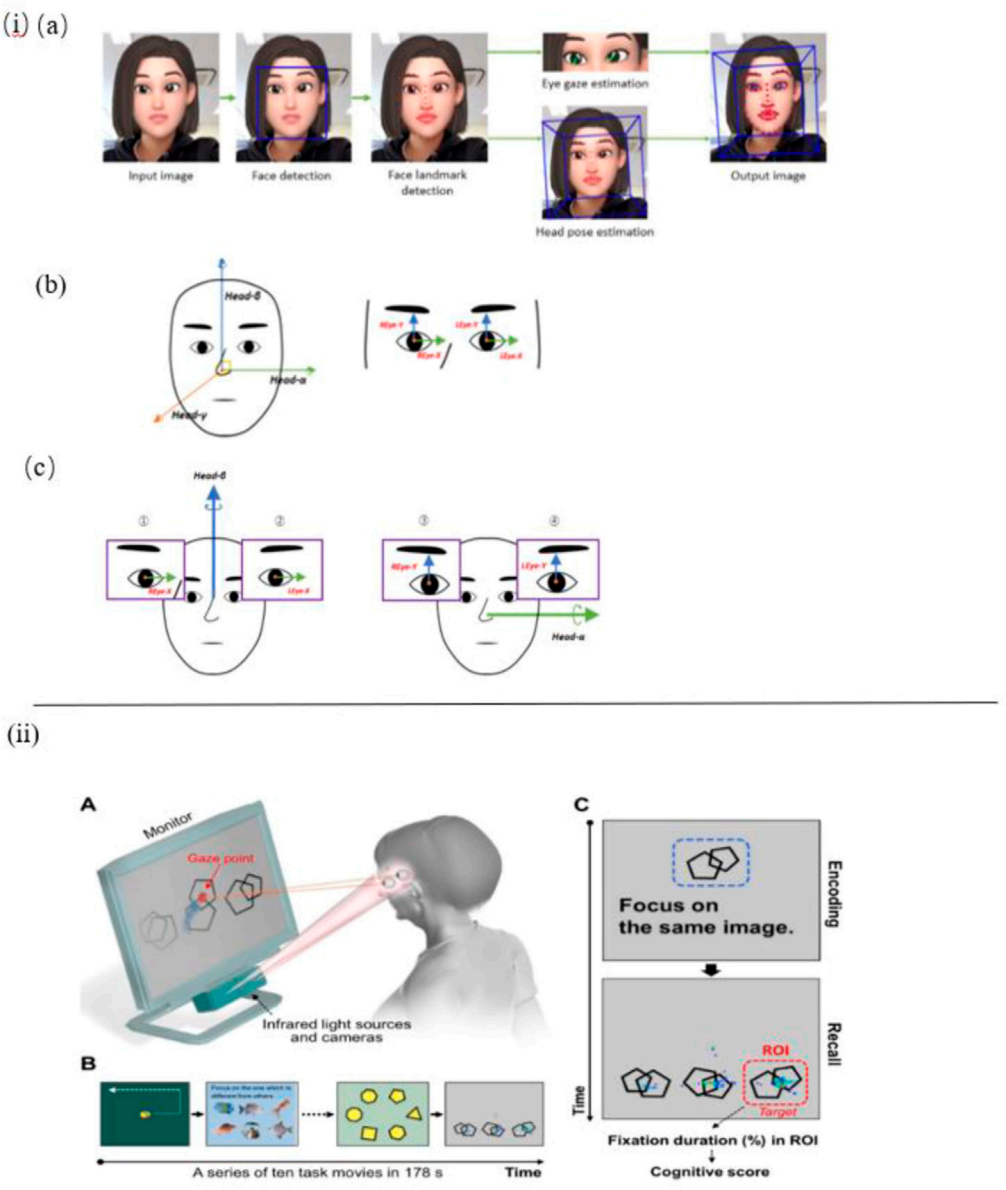
i) **(A)** Facial and eye movement extraction OpenFace 2.0. **(B)** Coordinate axes: face and eye. **(C)** A pair of axes was used to obtain the correlation coefficient: horizontal and vertical (Nam et al., 2020). ii) **(A)** Eye-tracking system. **(B)** Tasks for rapid cognitive assessment. **(C)** An example of a working memory task and representative gaze plots (Oyama et al., 2019).

Nam et al. constructed a sensor-based experimental setup to detect eye movement, head pose and their correlation (Figure 2I). To measure gaze behavior naturally, a camera rather than a wearable eye-tracker is equipped to record participants’ face video data. The findings suggest that AD patients’ eyes move with the head in the vertical orientation at the same time, whereas the healthy control did not show such behavior features. In conclusion, this eye and head movement behavior detection could be significantly useful in screening for AD (Nam et al., 2020).

In the work of Tadokoro et al. a novel infrared-based eye tracking camera is constructed to capture gaze points in the eye tracking test. Participants are asked to perform this 3-min image task displayed on a computer monitor with an audio explanation, the data recorded is used to analyze eye tracking scores and classify NC (Normal Control), MCI (Mild Cognitive Impairment), and AD subjects. As a result, it shows that eye tracking scores are correlative with mini-mental state examination (MMSE) scores and it is an effective and rapid method to detect early cognitive impairment (Tadokoro et al., 2021). In another work by Oyama et al., the very same eye-tracking device is used to perform a rapid cognitive assessment and show good diagnostic performance (Figure 2II) (Oyama et al., 2019).

In the study of Granholm et al., a handheld infrared sensor-based pupillometer is utilized to record pupil response evoked in cognitive tasks to detect biomarkers of early mild cognitive impairment (MCI) and AD risk prediction. By using these pupillometer devices MCI and cognitively normal (CN) participants can be differentiated, whose results are correlative with those in locus coeruleus examination (Granholm et al., 2017).

**FIGURE 3:**
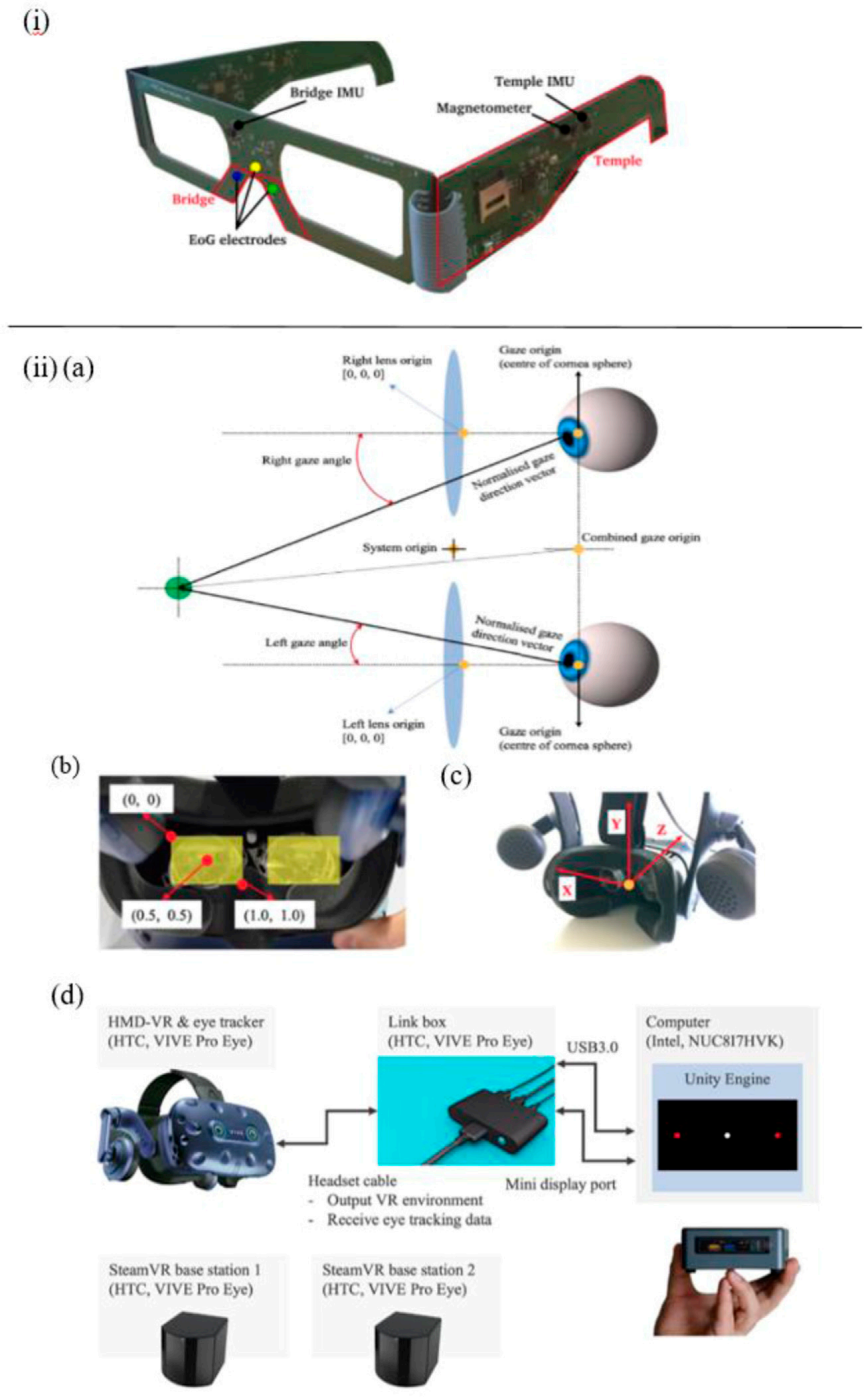
i) The primary parts of the glasses and an illustration of the employed sensors (Sciarrone et al., 2020). ii) **(A)** VIVE Pro Eye’s coordinated eye tracking system. **(B)** Coordinate system of pupil position data from the user’s perspective. **(C)** Coordinate system of gaze direction vector from user’s perspective. **(D)** Experimental technology for measuring saccadic eye movement utilizing HTC VIVE Pro Eye (Yu et al., 2020).

Apart from the non-wearable eye-tracking sensors mentioned above, various wearable eye trackers are applied in some works. For instance, Sciarrone et al. present a wearable sensor-based glasses to measure the Essential Tremor (ET) of the head and the number of Eye Blinks (EBs) simultaneously for early-stage AD patients (Figure 3I). These behavioral symptoms can be precisely detected, with an accuracy of 97% for ET, and Root Mean Square Error (RMSE) around 0.4 for EBs (Sciarrone et al., 2020).

In addition, Davis et al. reported a feasibility study of using virtual reality (VR) and eye tracking techniques in Way-finding tasks for Alzheimer’s disease research. In this study, wearable sensor-based eye tracking glasses with two cameras were applied to record video. One camera was used to record VR environments, which were projected on a 12-foot screen. Another one was used to capture subjects’ gaze data *via* the optical sensor device. In this way, 60% of eye-tracking videos of subjects who finished all trails were complete and usable, which proved the feasibility of using projected VR and eye tracking in large-scale wayfinding tasks for AD detection, but some limitations should be taken into consideration, such as joystick issues, simulation sickness, and calibration issues, *etc.* (Davis, 2021). Besides, compared with the eye tracker constructed in the projected VR environment mentioned in the study of Davis et al., Yu et al. employed a more immersive eye tracking device: a novel head-mounted VR device, in which a sensor-based eye tracker is embedded (Figure 3II). To be specific, biosensors installed in this VR device include steam VR tracking, accelerometer, gyroscope, proximity, interpupillary distance (IPD) sensor, near-infrared LED, and infrared camera. In their work, this wearable VR device is utilized to record saccade and other eye movement metrics in pro- and anti-saccade tasks. Consequently, the data analysis results indicate that it is able to measure saccadic eye movement parameters despite the technical limitations on time-linked parameters assessment (Yu et al., 2020).

### 2.3 Speech behavior detection of Alzheimer’s disease

Speech testing plays a critical part in the medical diagnosis of several neurodegenerative diseases (Veronica et al., 2017). Over the past years, studies have proved that a considerable percentage of AD patients suffer from vocal communication problems (Habash et al., 2012), and impairment in speech and language possibly be a powerful predictor of MCI and AD (Ammar and Ayed, 2020), some studies even suggested that speech behavior changes might be one of the earliest indicators of cognitive decline, frequently noticeable years before other cognitive impairments become apparent (Beltrami et al., 2018). The researchers analyzed a large number of language samples to extract the features, quantified the language automatically and combined it with machine learning classification methods to distinguish healthy controls from AD patients by detecting target language skills. Meanwhile, due to the non-invasive, convenient, and low-cost properties of speech analysis techniques (Pulido et al., 2020), various works have focused on conducting speech behavior analysis to obtain early indicators for the diagnosis of early Alzheimer’s disease (Samrah et al., 2013; Laske et al., 2015). For this purpose, various acoustic sensors have been employed to capture vocal signals from subjects’ speech.

**FIGURE 4:**
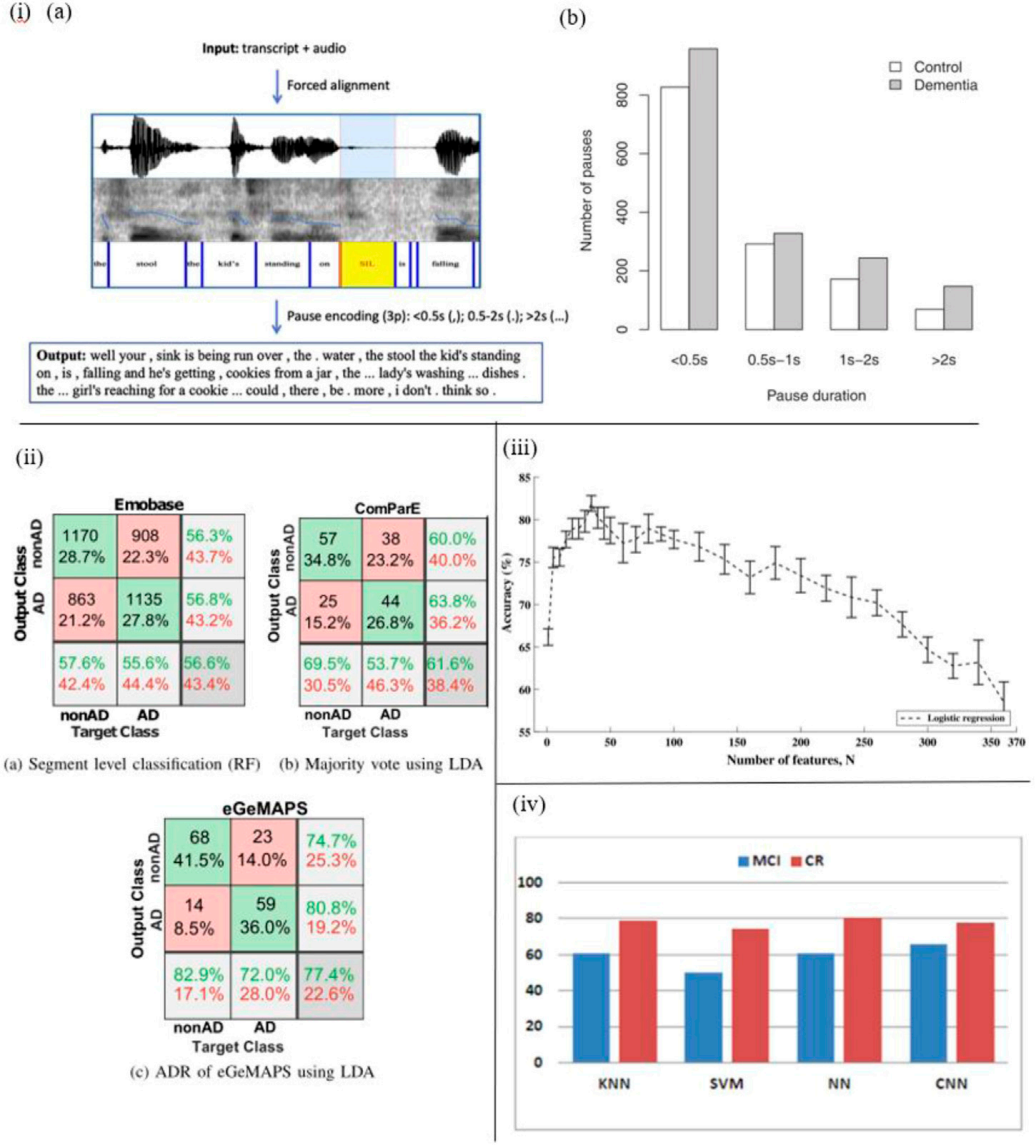
i) **(A)** The pause encoding process. **(B)** AD patients pause more often (in all duration bins) (95). ii) Confusion matrices that include the top outcomes from every experiment, as well as precision, recall, and total accuracy (93). iii) Accuracy and standard errors, divided by the N predictor characteristics employed (91). iv) CER (%) for classes and selected classifiers, including convolutional neural network, k-nearest neighbors, support vector machines, multilayer perceptron with L layers and N neurons (96).

For instance, López-de-Ipiña et al. proposed an automatic spontaneous speech analysis (ASSA) method for early Alzheimer’s disease diagnosis. Based on an automatic Voice Activity Detection (VAD) the recorded spontaneous speech and emotional speech analysis are conducted automatically (Figure 4I). By processing these vocal parameters, including duration, time domain, frequency domain, acoustic, and voice quality features, AD patients’ vocal features are identified (López-de-Ipia et al., 2013).

In another work by Fraser et al., with the help of sound sensor short narrative samples are extracted by performing a picture description task, and processed to form linguistic and acoustic variables. Moreover, these variables are used to train the machine learning classifier to classify AD and healthy subjects. Based on experiments, four linguistic features including semantic impairment, acoustic abnormality, syntactic impairment, and information impairment are proved to be useful in the recognition of AD, and the accuracy of this method reaches up to 81% (Fraser et al., 2015) (Figure 4III).

Laszlo et al. applied a dedicated automatic speech recognition (ASR) tool in automatic MCI screening. By analyzing the spontaneous speech of participants in two tasks, acoustic parameters are extracted automatically. In addition, machine learning algorithms are applied to assist distinguish MCI from healthy subjects. The results demonstrate that the speech tempo in the delayed recall task and the number of pauses in the question-answering task are the most notable differences between these two groups (Laszlo et al., 2018).

Haider et al. studied purely paralinguistic acoustic features of subjects’ spontaneous speech using a microphone and voice activity detection system. Based on this, four feature sets, e.g., Emobase, ComParE, eGeMAPS, and MRCG functionals are extracted and processed for AD detection with Machine Learning and Active Data Representation (ADR) methods. As a result, this work shows that such comprehensive acoustic feature sets of spontaneous speech are correlated with cognitive function, and might contribute to AD screening and diagnosis (Haider et al., 2020) (Figure 4II).

In the work of Calzà et al., linguistic feature modifications caused by cognitive decline have been studied to detect Mild Cognitive Impairment (MCI) and dementia *via* Natural Language Processing (NLP) techniques. To this aim, an Olympus-Linear PCM Recorder LS-5 is employed to record speech data of subjects during the implementation of three spontaneous speech tasks. In addition, acoustical, rhythmical, lexical, morpho-syntactic features and readability domains are extracted automatically by means of created algorithms, and then automatic classifiers are trained to discriminate between healthy control and MCI subjects (Figure 4III). The findings suggest that this automatic diagnosis system is able to distinguish healthy subjects from MCI (high F1 score, approximately 75%) (Calzà et al., 2021). The same recorder (Olympus-Linear PCM Recorder LS-5) has been applied by Beltrami et al. to record spontaneous speech during three tasks designed for 96 participants. As a result, many speech features, such as acoustic lexical and syntactic parameters are transcribed and analyzed by Natural Language Processing (NLP) to discriminate between healthy controls and cognitively impaired participants. The results indicate that this method might be a promising tool to recognize early-stage cognitive deficits (Beltrami et al., 2018).

Besides, several researchers studied disfluencies and pauses in speech to detect cognitive impairment (Yuan et al., 2021) (Figure 4I). López-de-Ipiña proposed an automatic analysis method of speech and disfluencies to assist MCI diagnosis. For this purpose, 40 speech samples from the MCI group and 60 from the control group are recorded by a sensor-based sound recorder, and automatically segmented into disfluencies *via* the VAD algorithm. Employing non-linear multi-feature modeling and deep learning approaches, the experiment shows hopeful results and provides a novel research direction (Lopez-De-Ipina et al., 2017) (Figure 4IV). Similarly, Yuan et al. studied disfluencies and language problems in Alzheimer’s Disease as well, by using fine-tuning Transformer-based pre-trained language models, e.g., BERT and ERNIE. In this work, 108 speakers’ speech samples in the training set and 48 speakers’ speech samples in the test set are recorded by recorders. As a result, **89.6%** accuracy on the test set of the Alzheimer’s Dementia Recognition through Spontaneous Speech has been achieved and the conclusion is drawn: AD patients speak *uh* more frequently than speak *um* (Yuan et al., 2020).

### 2.4 Multimodal detection of Alzheimer’s disease

In the studies mentioned above, most of the sensors have been used in isolation to detect behavior parameters. However, every single sensor has its merits as well as demerits, they cannot measure all the metrics needed alone (Alberdi et al., 2016). Therefore, a technology that can fuse all sensors is getting more attention in this field (Yang et al., 2022). Recently, the multi-sensor detection model is gaining more popularity in behavior detection (Hoque et al., 2015; Alberdi et al., 2016). Many studies have revealed that it is feasible to obtain comprehensive information on patients’ activity behavior in an indoor or experimental environment, based on the dense sensing system and sensor fusion technology (Abe et al., 2015; Stavropoulos et al., 2015; Chen et al., 2019). Thus, this strategy greatly improves the efficiency of patients’ behavior detection and has a more effective analyzing mechanism in assessing patients’ activity behavior with variable intensities compared to the conventional method (Deep et al., 2020).

As a result, in various indoor environments, multimodal detection sensors have been deployed to record and assess multiple behavioral signals of AD patients. For example, Jonell et al. proposed a multimodal capture method to detect AD patients’ behavior in a real clinical environment by using nine sensors, which include three smartphone cameras, a tablet, an eye tracker, a microphone array, a health wristband, a thermal camera, and an overview camera. To minimize distraction to the subjects and ensure the ecological validity of the recorded data, most of the installed sensors except for the health wristband are non-wearable. With their help, multimodal behavioral data are collected, such as facial gestures recorded by the patient camera (Smartphone Camera), gaze and pupil dilation by Eye Tracker, voice quality (breathiness, vocal strength), pauses, speech rate by Microphone Array, pen movement and pen pressure by Tablet, thermal emission data by Thermal Emission Camera, heart rate, galvanic skin response and acceleration by Health Wristband in clinical condition. In this way, the clinical feasibility of this sensor system is demonstrated by relating these digital biomarkers to traditional clinical assessment methods and established biomarkers (Jonell et al., 2021).

**FIGURE 5:**
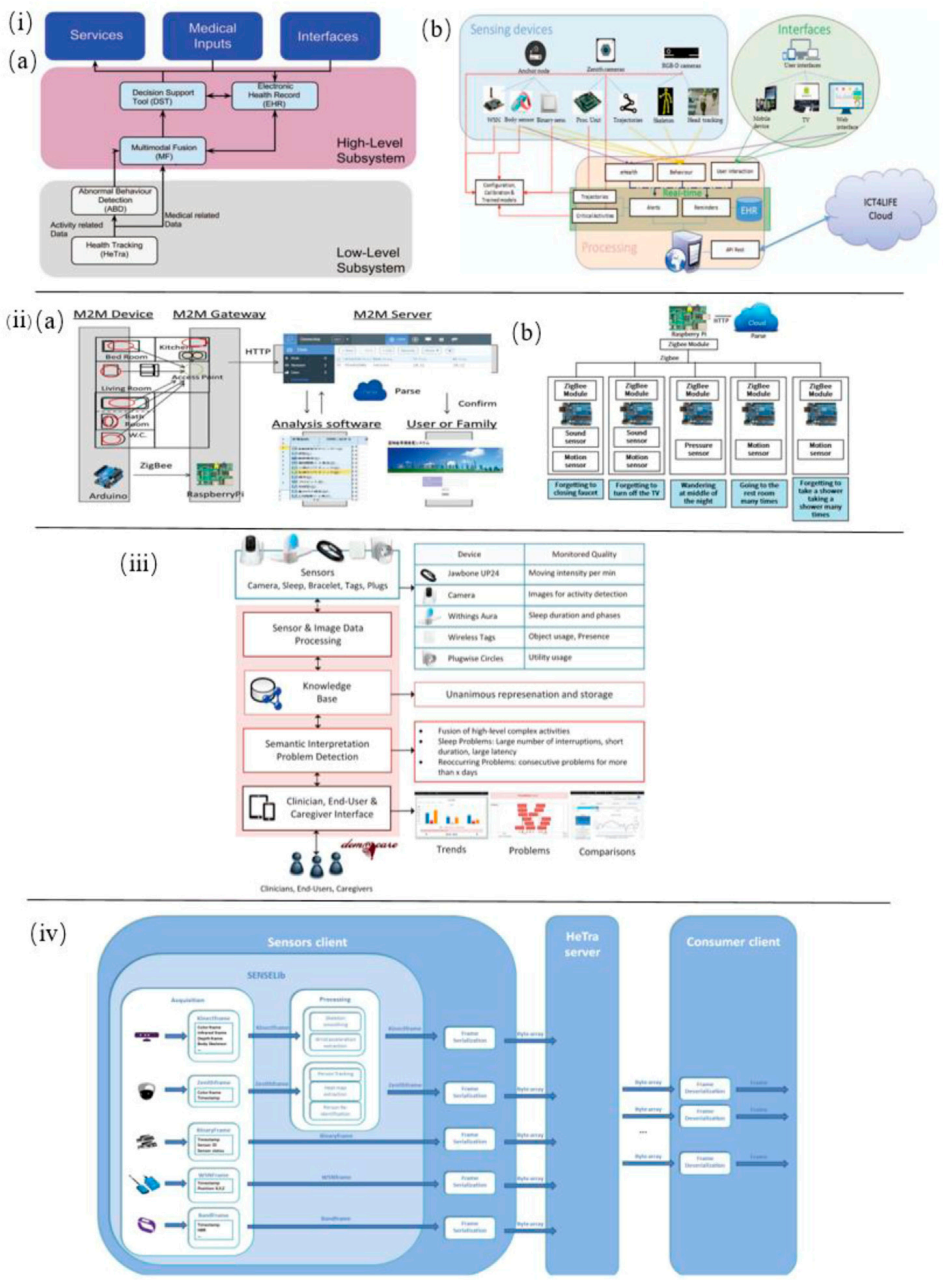
i) **(A)** Overview of the complete system architecture. **(B)** Low-level subsystem architecture for sensor-based health tracking (Alvarez et al., 2018). ii) **(A)** Schematic of the M2M System. **(B)** M2M device gateway (Ishii et al., 2016). iii) Architecture of the smart home monitoring system (Lazarou et al., 2016). iv) Sensor-based HeTra subsystem architecture overview (Alvarez et al., 2017).

In addition, Alvarez et al. developed a novel multimodal sensing system to capture and analyze AD patients’ physical abnormal behavior in daily motion. In this system various sensors are employed, including multisensory smart bands to offer blood pressure and skin temperature, an accelerometer, gyroscope, and magnetometer to capture motion data, a Binary sensor to detect whether doors and drawers are open, an RGB-D camera (Kinect) to collect deep motion data, a Zenith camera to record 360-degree panoramic view and Wireless sensor network (WSN) anchor or beacons to extract radio signals from wearable devices (Figure 5I). Therefore, based on these technical approaches, this system integrated with Internet of things devices and user interactions is able to provide automatic, distant monitoring of AD patients (Alvarez et al., 2018). In another work by Alvarez et al., the very same sensor-based devices were integrated into an ICT4LIFE platform to capture visual, motion and depth data of AD patients’ abnormal behavior (Figure 5IV ). Based on these data, behavior patterns are recognized and reported to the interested party to take appropriate actions (Alvarez et al., 2017).

Ishii et al. proposed a sensor network system toward the early detection of dementia for elderly people living alone. Inside their house, various sensors were installed to identify behaviors indicating initial symptoms of dementia. With the help of the M2M (Machine-to-Machine)/IoT (Internet of Things) platform these sensors’ data are recorded and analyzed for diagnosis of dementia. In this system, sensors such as sound sensor, pressure sensor, and motion sensor are applied to determine whether forgetting to close a faucet or turn off the TV, wandering in the middle of the night, going to the restroom many times, forgetting to take a shower and taking a shower many times (Figure 5II) (Ishii et al., 2016).

Zhi et al. studied the elderly’s behavior to predict the risk of Alzheimer’s disease by using IoT room sensors for location capture. Based on this, behavioral analysis models are developed to detect the three main variables of elderly behavior: sleeping patterns, excess active levels, and repetitive actions. In this work, 20 elderly people living independently participated in this 6-months-period experiment, and their accommodations five sensors are deployed. The experimental results show these behavior variables detected by IoT sensors are useful in predicting the early symptoms of potential Alzheimer’s disease (Zhi et al., 2017).

Lazarou et al. developed a multi-sensor-based intelligent home monitoring system for the elderly with cognitive deficits (Figure 5III). Data on sleep behavior and physical behavior of daily living have been collected and visualized. The results suggest that REM sleep in sleep quality is a key indicator to assess cognitive status among the detected abnormalities (Lazarou et al., 2016).

**FIGURE 6:**
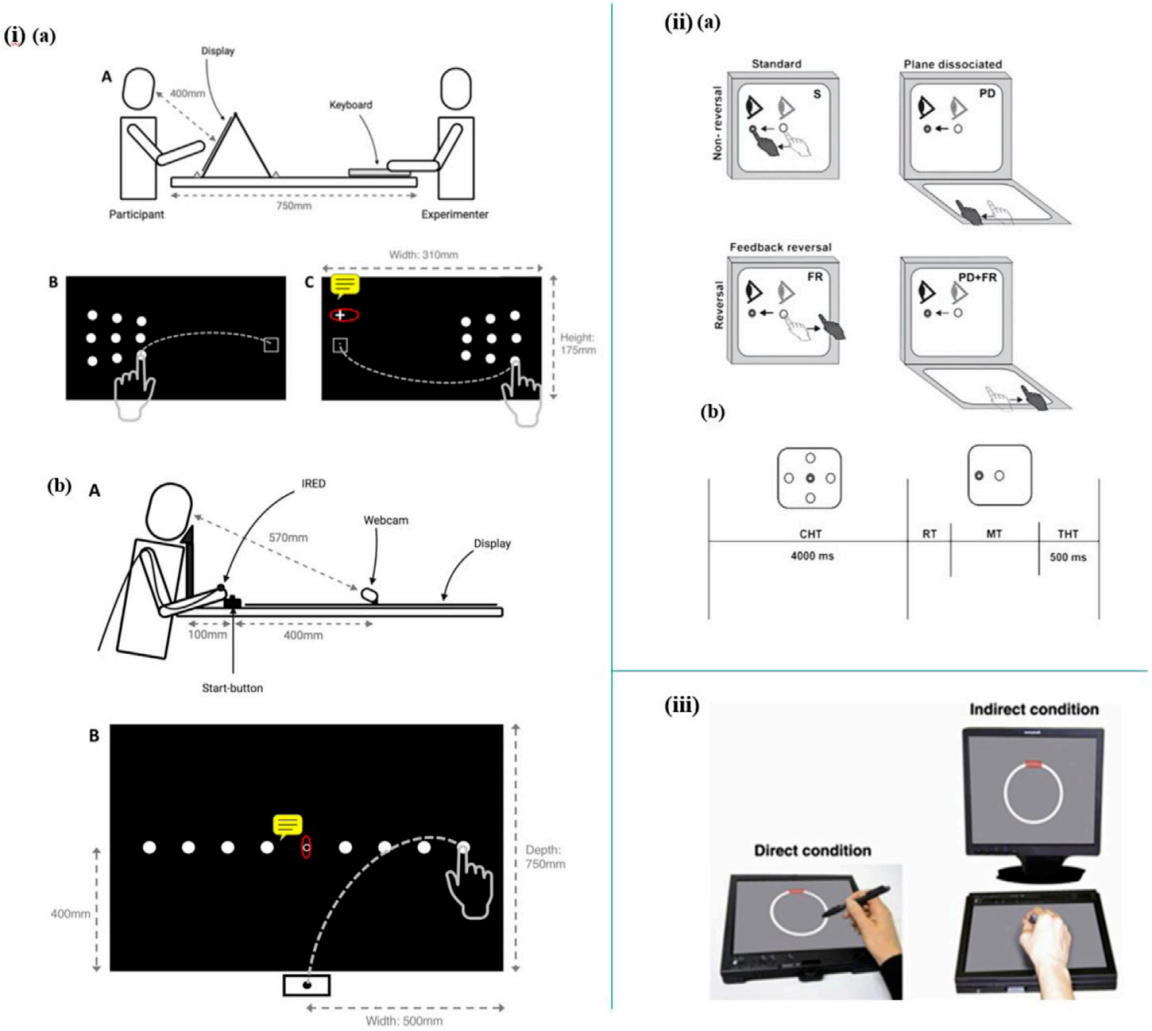
i) **(A)** Schematic diagram of lateral reaching task. **(B)** Schematic diagram of radial reaching task (Mitchell, Rossit, Pal, Hornberger, Warman, Kenning, Williamson, Shapland, McIntosh). ii) **(A)** Schematic diagram illustrating the four experimental conditions. **(B)** Trial timing (Hawkins and Sergio, 2014). iii) Setup of the circle-tracing task (Kirsty et al., 2017).

Apart from indoor living environments, multi-sensors have been utilized in specific experimental tasks. For instance, Staal et al. introduced a novel non-invasive, and time-saving method to predict Mild Cognitive Impairment (MCI) and Alzheimer’s Disease (AD) by detecting visuomotor network dysfunctions as potential biomarkers. In this study, three eye tasks and five eye-hand tasks were executed with the help of a touchscreen to display the stimulus, a head-mounted infrared eye-tracking system to record eye movement data, and an infrared motion capture system to record hand movements data. The data processed suggest that eye-hand tasks perform better in accuracy, sensitivity, and specificity than eye tasks to classify control, MCI, and AD. In addition, visuomotor features are potential biomarkers to predict MCI and AD (Staal et al., 2021). Moreover, visuomotor behavior has also been shown to be impaired in the early stage of Alzheimer’s Disease by Hawkins et al. by employing a touchscreen to perform four visuomotor tasks (Figure 6II) (Hawkins and Sergio, 2014).

Mitchell et al. reported a novel research on peripheral reaching movements in AD and MCI. In the peripheral reaching tasks, participants are required to reach stimuli displayed in their peripheral vision. Meanwhile, the reaching movements data were tracked by using an infrared motion-tracking camera, which sampled the 3D position of infrared-emitting diodes (IREDs) taped to subjects’ fingernails (Figure 6I). The results show that movement features observed in these peripheral reaching tasks are not common behavior markers of AD. However, the prolonged reaching time is possibly correlated with visuomotor impairments and may indicate a potential risk of AD and MCI (Mitchell, Rossit, Pal, Hornberger, Warman, Kenning, Williamson, Shapland, McIntosh).

Kirsty et al. established a sensor-based setup to explore whether the deficits in visuomotor integration behavior are early-stage symptoms of cognitive impairment of autosomal dominant familial AD (FAD). In this work, a sensor-based tablet screen and a stylus are employed in 12 circle-tracing tasks, which include six direct tracing tasks and six indirect tracing tasks (Figure 6III). As a result, the analyzed data suggest that measurement of visuomotor integration deficits could be a promising approach to predict FAD (Kirsty et al., 2017).

Additionally, in the work of Shinkawa et al., the combination of speech and gait behavioral data has been studied with the help of three microphones (a throat microphone, a lavalier microphone, and the iPad’s internal microphone) and a motion capture system. Based on the collected multimodal behavior data: voice and body position data, healthy control and MCI group have been discriminated with an accuracy of 82.4%, increased by 5.9% than in single modality condition (Shinkawa et al., 2019).

## 3 Conclusion and future perspectives

In this review, the state-of-the-art applications of sensors toward behavior detection in the diagnosis of Alzheimer’s disease have been overviewed from four different perspectives: body motion behavior detection, eye movement behavior detection, speech behavior detection, and multi-behavior detection. The specific examples of these applications have been tabulated in Tables 1–4 in detail. As can be seen from the above table, the results of the multimodal integrated test is the most accurate method in the diagnosis of Alzheimer’s disease and is usually able to distinguish between normal and abnormal behaviours with good adaptability, the eye-movement behavioural tracking approach requires an experimental environment and has high requirements for the testing environment, but has a good future as it can reach an accuracy of up to 97%. Lastly, physical behavioural monitoring, with its good correlation between experimental results and those of traditional tools and low requirements for the diagnostic environment, is a good diagnostic tool for Alzheimer’s disease.

**TABLE 1:** List of sensor-based body motion behavior detection toward AD diagnosis.

	Devices used	Sensor	Type of sensor	Parameter detected	Environment	Effectiveness	Subjects	Ref
Body motion behavior detection	Feet and waist-mounted devices	Inertial sensor (accelerometer, gyroscope)	Wearable, non-invasive	Gait, balance	Indoor experimental environment	Promising indicator for early diagnosis of AD	70 (21 AD, 50 HCs)	Hsu et al. (2014)
	Ankle-mounted device	Three-axes accelerometer	Wearable, non-invasive	Spatial trajectory	Indoor experimental environment	Classification accuracy: 91%	46 (23 AD, 23 HCs)	Kirste et al. (2014)
	Leg movement IoT device	Force sensor	Wearable, non-invasive	Walking speed, gait	Indoor experimental environment	Sensitivity: 95.9% Specificity: 94%	173 AD, 150 HCs	Varatharajan et al. (2017)
	SensorFoot (Foot-mounted sensor device)	9-axis inertial sensor (3-axis accelerometer, 3-axis gyroscope, 3-axis magnetometer)	Wearable, non-invasive	Kick	Indoor experimental environment	Good correlation with traditional approaches	15 (4 MCI, 11 HCs)	Fiorini et al. (2017)
	SensorFoot V2 (Foot-mounted sensor device)	9-axis inertial sensor (3-axis accelerometer, 3-axis gyroscope, 3-axis magnetometer)	Wearable, non-invasive	Kick	Indoor experimental environment	Good correlation with traditional approaches	49 (20 MCI, 29 HCs)	Fiorini et al. (2019)
	Wrist-mounted sensor-based device	Actigraph accelerometer	Wearable, non-invasive	Sedentary behavior, moderate-to-vigorous activity behavior	Indoor free-living environment	Good correlation with cognitive functioning	66 non-demented adults with down syndrome	Fleming et al. (2021)
	Wrist-mounted sensor-based device	Accelerometer	Wearable, non-invasive	Sedentary behavior	Indoor free-living environment	Good differentiation between AD, MCI and HCs	671	Lu et al. (2018)
	SmartTapestry (sensor-based tapestry)	Sensing units	non-wearable, non-invasive	upper limb articulation movement	Indoor experimental environment	Good correlation with traditional tool	-	Maselli et al. (2017)
	In-home wireless sensor system	Ambient sensors	non-wearable, non-invasive	Activities of daily living (ADL)	Indoor free-living environment	Differentiation accuracy:95%	10 dementia patients, 10 HCs	Urwyler et al. (2017)
	Head-mounted VR glasses, Kinect sensor device	Depth sensor	non-wearable, non-invasive	Body movement	Virtual environment	A promising solution for AD diagnosis	20	Montenegro and Argyriou, (2017)

**TABLE 2:** List of sensor-based eye movement behavior detection toward AD diagnosis.

	Devices used	Sensor	Type of sensor	Parameter detected	Environment	Effectiveness	Subjects	Ref
Eye movement behavior detection	Desk-mounted eye tracker	Infrared sensor	Non-wearable, non-invasive	Gaze, saccade, fixation	Lab experimental environment	Differentiation accuracy: 86%	-	Fraser et al. (2017)
	Camera	Imaging sensor	Non-wearable, non-invasive	Gaze, head pose	Lab experimental environment	Good differentiation	34 (17 mild AD, 17 HCs)	Nam et al. (2020)
	Eye tracking camera, computer monitor	Infrared sensor	Non-wearable, non-invasive	Gaze	Indoor experimental environment	Good correlation with MMSE	174 (52MCI, 70AD, 52 HCs)	Tadokoro et al. (2021)
	Handheld pupillometer device	Infrared sensor	Non-wearable, non-invasive	Pupil diameter	Indoor experimental environment	Good differentiation, good correlation with locus coeruleus examination	918	Granholm et al. (2017)
	Eye-tracking glasses	Image sensor, magnetometer, EOG	Wearable, non-invasive	Eye blink (EB), essential tremor of the head (ET)	Indoor experimental environment	accuracy of 97% for ET, and Root Mean Square Error (RMSE) around 0.4 for EBs	5	Sciarrone et al. (2020)
	Eye-tracking glasses	Optical sensor	Wearable, non-invasive	Gaze	Projected virtual environment	Relatively well-tolerated in older adults and AD patients	88 (38 AD, 50 HCs)	Davis, (2021)
	Head-mounted VR device	SteamVR tracking sensor, accelerometer, gyroscope, proximity, interpupillary distance sensor, infrared sensor	Wearable, non-invasive	Saccade, pupil diameter	Full-virtual environment	Effective assessment tool	7	Yu et al. (2020)

**TABLE 3:** List of sensor-based speech behavior detection toward AD diagnosis.

	Devices used	Sensor	Type of sensor	Parameter detected	Environment	Effectiveness	Subjects	Ref
Speech behavior detection	Microphone	Sound sensor	Non-wearable, non-invasive	Vocal feature：duration, time domain, frequency domain, acoustic and voice quality features	Lab experimental environment	Satisfactory results, a promising approach for early diagnosis and discrimination of AD	70 (20 AD, 50 HCs)	López-de-Ipia et al. (2013)
	Voice recorder	Sound sensor	Non-wearable, non-invasive	Acoustic abnormality, semantic impairment, syntactic impairment, and information impairment	Lab experimental environment	Classification accuracy: 81%	264 (167AD, 97 H C)	Fraser et al. (2015)
	Digital voice recorder, tie clip microphone	Sound sensor	Non-wearable and wearable, non-invasive	Hesitation ratio, speech tempo, articulation rate, length and number of silent and filled pauses, length of utterance, pause-per-utterance ratio	Lab experimental environment	Classification accuracy: 78.8%	84 (36HCs, 48 MCI)	Laszlo et al. (2018)
	Microphone, voice activity detection system	Sound sensor	Non-wearable, non-invasive	Emobase, ComParE, eGeMAPS and MRCG functionals	Lab experimental environment	Classification accuracy: 78.7%	—	Haider et al. (2020)
	Voice recorder	Sound sensor	Non-wearable, non-invasive	Acoustical, rhythmical, lexical, morpho-syntactic features and readability domains	Lab experimental environment	Classification accuracy: 75%	96 (48HCs, 48 impaired subjects)	Calzà et al. (2021)
	Voice recorder	Sound sensor	Non-wearable, non-invasive	Acoustic, rhythmic, lexical and syntactic parameters *etc.*	Lab experimental environment	A promising tool to recognize cognitive deficits in early stage	96 (48HCs, 48 cognitively impaired participants)	Beltrami et al. (2018)
	Voice recorder	Sound sensor	Non-wearable, non-invasive	alse starts, repeated or re-started phrases, repeated or extended syllables, grunts or non-lexical utterances and instances of speakers correcting their slips of the tongue or mispronunciations	Lab experimental environment	Hopeful approach for automatic detection of MCI	100 (40MCI, 60HCs)	Lopez-De-Ipina et al. (2017)
	Voice recorder	Sound sensor	Non-wearable, non-invasive	Word frequency and uh/um, unfilled pauses	Lab experimental environment	Recognition accuracy: 89.6%	156 (78AD, 78HCs)	Yuan et al. (2020)

**TABLE 4:** List of sensor-based multi-behavior detection toward AD diagnosis.

	Devices used	Sensors	Type of sensor	Parameter detected	Environment	Effectiveness	Subjects	Ref
Multi-behavior detection	Three smartphone cameras, a tablet, eye tracker, microphone array, health wristband, thermal camera, and an overview camera	Image sensor, touch sensor, infrared sensor, sound sensor, heart rate sensor, accelerometer, skin electric transducer, thermal sensor	Non-wearable and wearable, non-invasive	Facial gestures, gaze and pupil dilation, voice quality (breathiness, vocal strength), pauses, speech rate, pen movement and pen pressure, thermal emission data, heart rate, galvanic skin response and acceleration	Real clinical environment	Good feasibility and effectiveness to improve the clinical assessment of early dementia	25 patients	Jonell et al. (2021)
	Multisensory smart bands, Binary sensor, RGB-D camera (Kinect), Zenith camera, Wireless sensor network (WSN) anchor or beacons	Blood pressure sensor temperature sensor, accelerometer, gyroscope and magnetometer, image sensor, ambient sensor, motion sensor	Non-wearable and wearable, non-invasive	Blood pressure and skin temperature, motion data, radio signals, ambient information	Indoor living environment	Good performance in discrimination between normal and abnormal behavior	700 sample trajectories	Alvarez et al. (2017)
	M2M(Machine-to-Machine)/IoT (Internet of Things) platform	Sound sensor, pressure sensor and motion sensor	Non-wearable, non-invasive	Abnormal behavior, ambient information	Indoor living environment	Good adequacy of detection and usability in detecting AD risk	Pseudo patients	Ishii et al. (2016)
	IoT sensors	Five room sensors	Non-wearable, non-invasive	Sleeping patterns, excess active levels and repetitive actions	Indoor living environment	A potential early diagnosis of AD and potential benefits of IoT sensors in studying the behavior of elderly	20 elderly people living alone	Zhi et al. (2017)
	Multi-sensor-based intelligent home monitoring system: camera, bracelet, wireless tag sensor, aura, plug	Image sensor, motion sensor	Non-wearable and wearable, non-invasive	Sleep behavior and physical behavior of daily living	Indoor living environment	A necessary tool for clinicians to efficiently assess participants’ abnormalities	4 subjects	Lazarou et al. (2016)
	A head-mounted eye-tracking system, a motion capture system	Infrared sensor, motion sensor	Wearable, non-invasive	Eye movements, hand movements, visuomotor feature	Lab experimental environment	Potential biomarkers to predict MCI and AD, discrimination accuracy is equivalent to the existing CSF and MRI biomarkers	96 subjects	Staal et al. (2021)
	Camera, index fingernails taped infrared-emitting diodes (IREDs)	Infrared sensor, motion sensor	Non-wearable and wearable, non-invasive	3D position hand movement, eye movement	Lab experimental environment	Prolonged visually guided movements indicate subtle visuomotor impairment in AD	51 (10MCI, 17AD, 24HCs)	(Mitchell, Rossit, Pal, Hornberger, Warman, Kenning, Williamson, Shapland, McIntosh)
	Tablet screen and a stylus	Touch sensor	Non-wearable, non-invasive	Tracing speed (number of rotations) and error rate (number of deviations outside the annulus per rotation	Lab experimental environment	A promising approach to predict FAD	31 participants	Kirsty et al. (2017)
	Throat and lavalier microphones, a motion capture system	Sound sensor, motion sensor	Wearable, non-invasive	Speech and gait behavior	Lab experimental environment	Discrimination accuracy: 82.4%	34 (15 MCI, 19 HCs	Shinkawa et al. (2019)

At this stage, there are three main methods to detect Alzheimer’s disease: 1. The neurologist assesses the target’s cognitive status using a special assessment form.; 2. Through puncture sampling to check for two typical biomarkers in the cerebrospinal fluid such as TAU protein and beta amyloid (Hyman et al., 2012) 3. Through magnetic resonance plus angiography of the head to detect neurodegeneration, atrophy, etc (Braak and Braak, 1994). All of these methods require specialist personnel or specialist instruments, even for the working environment, and some of the results can be subjective to the level of expertise of the assessor, making the development of a variety of wearable, portable, small, non-intrusive sensing devices an urgent challenge.

For the detection of physical behaviour, the sensors can often be worn for longer periods of time and observed for longer periods of time, but the relationship between prolonged physical activity and cognitive function still requires further research, as there are relatively more factors that can affect physical activity and more interfering factors that cannot be accurately ruled out (Fleming et al., 2021).

Traditional screen-based eye-tracking technology usually requires the head to be immobilised in order to obtain information about eye movements and visual attention, which restricts the subject’s movement and reduces the experience of using the device. The new VR-style device is relatively more tolerable for testing equipment, so that the subject’s movement is no longer restricted and can better cooperate with the test (Davis, 2021).

Language impairment is present in a wide range of neurodegenerative diseases and can be assessed by natural language tests or by detecting speech signals in conversations *etc.* The two can be cross-checked and for the extraction of speech signals it is even possible to differentiate between differences caused by the primary disease, ageing and dementia, making the assessment results more accurate, but the level of speech is influenced by the speaker’s prior or unconscious, while environmental sounds can also influence speech Acoustic detection (Yang et al., 2022) (Coupland, 2007) (Chen et al., 2019). For people with different cultural and educational backgrounds, picture description tasks are not fully applicable and can affect the accuracy of clinical applications.

In general, non-invasive biosensors are favored in most recent behavior detection related studies of AD diagnosis, considering the safety and comfort factors. Besides, both wearable and non-wearable biosensors have been extensively applied in different research settings, respectively. Specifically, biosensors employed in body motion detection are mainly wearable biosensors, such as foot-mounted sensors (e.g., SmartWalk) and wrist-mounted sensors (wrist-mounted accelerometer), which allow subjects to move their body at different positions as required in an experiment, and record their physical behavior data in real-time. Meanwhile, the biosensors used for eye movement behavior detection include both wearable (e.g., eye-tracking glasses) and non-wearable (e.g., desk-mounted eye-tracker), because in some motionless experiment settings, to obtain more natural eye movement behavior data non-wearable sensors are favored (Nam et al., 2020), while some body motion required tasks or virtual experimental environments wearable sensors are preferred devices (Yu et al., 2020; Davis, 2021). However, when it comes to speech behavior and multi-behavior detection, the majority of biosensors introduced are non-wearable categories, to reduce interference with the subjects, which may include, for instance, desk-placed voice recorders, Kinect (depth) sensors, smartphone cameras, and microphone, *etc.* (Jonell et al., 2021). Therefore, they are mostly unobtrusively installed in the surrounding environment.

In the future, several more prominent trends will emerge in the use of behavior detection sensors in AD diagnostics. Firstly, more sensor-based behavior detection experiments will be integrated into the Internet of Things (IoT) platform. Significant advances in IoT technology have been achieved in medical detection, and it will be more widely used in behavior detection due to its great advantages (Javdani and Kashanian, 2017). IoT systems can generate and transmit signals to professionals, which allows them to gather behavior data in real-time and to continuously monitor and assess patients’ behavior remotely 24/7. Based on this technology, AD patients, especially those elderly patients living alone can be monitored continuously, their abnormal behavior can also be detected at any time. In this way, it provides a possibility to detect potential risks of disease among them at an early stage.

Secondly, multi-behavior sensing technology will be favored in various detection and monitoring experiment. Compare to the single modality approach, multimodal sensing has higher accuracy in AD assessment (Shinkawa et al., 2019). In the future, the development trend of behavior detection in AD diagnosis is to sense large-scale multimodal information based on dense-sensing system and sensor fusion technology (Gravina et al., 2016) and capture various digital markers from physiological signals, environmental information, and body motion data simultaneously. By collecting and analyzing such comprehensive data, behavior detection is a more promising method in AD diagnosis at an early stage.

Finally, Virtual Environments (VEs) and Virtual Reality (VR) technology will gain more popularity in medical diagnosis and rehabilitation therapies owing to the advance in state-of-the-art technology in computer and communication science (Konstantinidis et al., 2017). As a result, based on immersive technology and Human-Computer Interaction (HCI), novel Alzheimer’s screening tests in virtual environments has been introduced and is drawing more attention to AD diagnosis (Montenegro and Argyriou, 2016). This is because VR technology makes it possible to provide an alternative solution to the traditional cognitive testing approach in AD, due to its safe, immersive experimental scenarios and the possibility of manipulating reality (Montenegro et al., 2020). Up to now, the virtual environment applied in AD assessment is mainly classified into two categories: full-immersive (Yu et al., 2020) and semi-immersive (Davis, 2021). Among them, the most commonly used sensor-based device is head-mounted VR, which can be embedded in an eye-tracking module to detect eye movement and connected with a Kinect (depth) sensor to detect body motion behavior. In the future, more multi-sensors will be added to it to capture a greater variety of behavioral signals, such as sound and physiological information, *etc.*
